# Monitoring quality indicators for the Xpert MTB/RIF molecular assay in Ethiopia

**DOI:** 10.1371/journal.pone.0225205

**Published:** 2019-11-12

**Authors:** Abebaw Kebede, Dereje Beyene, Bazezew Yenew, Getu Diriba, Zemedu Mehamd, Ayinalem Alemu, Misikr Amare, Gobena Ameni

**Affiliations:** 1 Ethiopian Public Health Institute, Addis Ababa, Ethiopia; 2 Department of Microbial, Cellular and Molecular Biology, College of Natural and Computational Sciences, Addis Ababa University, Addis Ababa, Ethiopia; 3 Aklilu Lemma Institute of Pathology, Addis Ababa University, Addis Ababa, Ethiopia; University of Ghana College of Health Sciences, GHANA

## Abstract

**Introduction:**

In Ethiopia, >300 GeneXpert instruments have been deployed for tuberculosis (TB) testing using the Xpert MTB/RIF cartridge. Implementing quality indicators is necessary for monitoring and evaluating the quality of Xpert MTB/RIF diagnostic services.

**Objective:**

To assess the use of quality indicators for the Xpert MTB/RIF molecular assay in Ethiopia and to compare the findings with the predefined targets described in the literature.

**Methods:**

Clinical specimens collected from patients with suspected TB were subjected to Xpert MTB/RIF testing at the National TB Reference Laboratory (NTRL) between January and December 2018. Data were collected from GeneXpert software and Laboratory Information System (LIS) databases. Quality indicators were calculated and analyzed. Bivariate and multivariate analyses were performed using SPSS software version 20 (SPSS Inc., Chicago, Illinois, USA).

**Results:**

Of the 2515 specimens tested, 2274 (90.4%) had successful test results; 18.2% were positive for *Mycobacterium tuberculosis* (MTB). Among MTB positives (n = 413), 4.8% and 1.0% were rifampicin (RIF)-resistant and RIF-indeterminate cases, respectively. Unsuccessful results were 241 (9.6%); 8.9% of the total number of tests were errors, 0.04% had invalid results and 0.6% ‘no result’. The most frequent error was probe check failure (error 5007). Instrument module A4, B2, B3, C3, and D3 (p<0.05) and tester experience (p<0.05) had a statistically significant association with errors in multivariate analysis. Additional 42 MTB cases (9.2% of the total cases) were detected among unsuccessful results by follow-up tests. Sixty-four percent of the initial test results were released within the turnaround time (TAT) ≤24 hours.

**Conclusion:**

Most of the quality indicators for the Xpert MTB/RIF molecular assay were maintained within the targets. However, the error rate and TAT were out of the targets. Defective modules and lacking experience were the factors affecting successful test outcomes.

## Introduction

The World Health Organization (WHO) has endorsed use of the Xpert MTB/RIF assay (Cepheid, Sunnyvale, CA) for the detection of *Mycobacterium tuberculosis* (MTB) and associated rifampicin resistance near the point of care, facilitating rapid diagnosis of tuberculosis (TB) and drug resistant TB (DR-TB) in adults and children with presumptive pulmonary and extrapulmonary TB [1–3]. As a result, Xpert MTB/RIF testing is being scaled up to all over the world. In Ethiopia, over 300 GeneXpert instruments have been deployed in different health facilities since 2012. The national Xpert MTB/RIF implementation guideline-recommended applying the Xpert MTB/RIF technology in high-risk groups for DR-TB, HIV seropositive individuals, children (<14 years of age), and patients with presumed extrapulmonary TB [4]. In August 2018, the Ethiopian National TB Control Program recommended that the Xpert MTB/RIF assay be used for testing on specimens from all presumptive TB patients irrespective of risk for DR-TB, HIV status, and age of the patient if Xpert MTB/RIF is accessible [5]. However, the quality of Xpert MTB/RIF testing has to be ensured in order to maximize the benefits for patient care and rapid diagnosis.

Xpert MTB/RIF is an automated molecular assay that simultaneously detects MTB and its resistance to rifampicin in less than two hours, uses heminested real-time polymerase chain reaction (PCR) assay to amplify MTB specific *rpoB* gene sequence [6]. The assay uses five different probes (A, B, C, and D) with molecular beacons in detecting mutations within the rifampicin-resistance determining region. The test integrates sample processing and PCR in a disposable plastic cartridge containing all reagents required for mycobacterial lysis, DNA extraction, amplification, and detection [2].

Quality indicators measure the degree to which a set of inherent characteristics fulfill performance requirements [7]. Moreover, the indicators validate how well the laboratory meets the requirements of the quality of the testing processes (pre-analytical, analytical, and post-analytical phases). According to the ISO 15189 standard, the laboratory should establish quality indicators for systematically monitoring and evaluating the laboratory’s contribution to patient care [7]. The quality indicators should be periodically reviewed to ensure their continued appropriateness.

For TB culture test, the quality indicators have been comprehensively reported in different mycobacteriology laboratories [8–10]; however, quality indicators for Xpert MTB/RIF assessment was made in Ethiopia and thus was not yet reported. We implemented the quality indicators recommended for Xpert MTB/RIF at the National TB Reference Laboratory (NTRL) of Ethiopia. Targets were set for the quality indicators from guidelines [11, 12] or literature [13–18] (Table 1). Any observed changes outside of the targets require an investigation for identifying potential causes. Therefore, the objective of this study was to assess the quality indicators for Xpert MTB/RIF molecular assay on the basis of the predefined targets described in the literature.

**Table 1:** Quality indicators implemented to monitor the performance of Xpert MTB/RIF molecular assay in Ethiopia.

Indicator	Numerator	Denominator	Target
Indicator 1: Percentage of specimens reported as MTB detected (MTB positivity rate)	Number of specimens reported as MTB detected RIF resistance not detected, RIF resistance detected, and RIF indeterminate in 1 month	Total number of specimens tested in 1 month	13.42–24.61%[13–18]
Indicator 2: Percentage of specimens reported as MTB detected; rifampicin resistance detected (RIF resistance rate)	Number of specimens reported as MTB detected RIF resistance detected in 1 month	Total number of MTB detected in 1 month	5.8% (2.8–8.4%)[12]
Indicator 3: Percentage of specimens reported as MTB detected; rifampicin indeterminate (RIF resistance indeterminate rate)	Number of specimens reported as MTB detected RIF indeterminate in 1 month	Total number of MTB detected in 1 month	8.9%[17] 13.6%[13]
Indicator 4: Percentage of specimens with error results (Error rate)	Number of specimens with error results in 1 month	Total number of specimens tested in 1 month	<3%[11]
Indicator 5: Percentage of specimens with invalid results (Invalid rate	Number of specimens with invalid results in 1 month	Total number of specimens tested in 1 month	<1%[11]
Indicator 6: Percentage of specimens with “no results” (No result rate)	Number of specimens with “no results” in 1 month	Total number of specimens tested in 1 month	<1%[11]
Indicator 7: Percentage of Xpert MTB/RIF results reported within TAT for results (Within TAT rate)	Number Xpert MTB/RIF results reported within the target TAT (2–24hrs) for results	Total number of Xpert MTB/RIF results reported	90%[11]

## Materials and methods

### Sample collection and processing

For patients with suspected pulmonary TB, a single spot sputum specimen (a minimum of 1.0ml) was collected using a sterile 50ml Falcon tubes following proper patient instruction at the reception unit of Ethiopian Public Health Institute (EPHI). Non-respiratory specimens were collected aseptically using an appropriate procedure by specially trained clinicians and the specimens transferred into a sterile 50ml Falcon tube and sent to NTRL of EPHI. Specimens were processed as previously described and as per manufacturer’s recommendations [2, 19]. In the case of unsuccessful (error, invalid, and no result) and RIF resistance indeterminate test results, repeating a test was carried out using the leftover Sample Reagent (SR)-treated sample within 12 hours (if kept in a refrigerator at 2–8°C) or from a newly collected specimen. Sixteen modules GeneXpert instrument was utilized for sample testing during the testing period. The GeneXpert® Dx Version 4.7b Software was used for Xpert MTB/RIF testing.

### Test related data collection and analysis

For each specimen, the following information was collected: laboratory identification number, referring health facility, specimen type, specimen quality (in case of sputum), specimen volume, Xpert MTB/RIF test result, error code, reagent lot, dates and times of specimen collected, tested and reported, dates and times of retesting (in the initial test was unsuccessful; error, no result and invalid, and indeterminate), tester identifier, and tester experience (<2yrs., 2–3 yrs., and >3 yrs.) in Xpert MTB/RIF testing. The details of test-specific errors were collected from the Errors tab of the View Results window. Each error codes were further defined based on code definitions in the GeneXpert Dx System Operator Manual[20]. The quality indicators; percentage of samples reported as MTB detected (Indicator 1), RIF resistant MTB (Indicator 2), RIF indeterminate MTB (Indicator 3), error (Indicator 4), invalid (Indicator 5), and ‘no result’ (Indicator 6), were calculated and analyzed as defined in Table 1. Turnaround time (the period between the specimen receipt and the test report released from the laboratory) (Indicator 7) was also calculated as one of the quality indicators. We analyzed the quality indicators by considering the initial test outcome only, but not the retesting results. The calculated value of the indicators was compared against the targets. Bivariate analysis was performed using SPSS version 20 (SPSS Inc., Chicago, Illinois, USA) to identify the associated causes among the possible factors for the indicators out of the limit or target. Multivariate analysis was performed using models that included a variable that was significate in the bivariate analysis (p ≤ 0.2). A p-value <0.05 was considered statistically significant. As this was a retrospective study using anonymous data, ethics approval not sought. All data were fully anonymized before accession.

## Results

### Demographic and clinical characteristics

A total of 2515 clinical specimens were collected from 2441 presumptive TB patients during the period between January 01, 2018 through December 31, 2018; 1895 (75.3%) were respiratory and 620 (24.7%) were non-respiratory specimens. The majority of the patients from whom specimens collected were male (57.9%); the median age of patients was 38 years (IQR, 27–54) (Table 2). The specimens were collected from patients found in the Addis Ababa City, the Capital of Ethiopia. The large majority of patients (96.1%) provided a single specimen; 66(2.7%) patients provided two specimens and 4 (0.2%) patients provide three specimens. Twenty-five patients (1.0%) submitted two or three clinical specimens from different sources or anatomical sites.

**Table 2:** Demographic and clinical characteristics of patients diagnosed with Xpert MTB/RIF molecular assay, 2018 (N = 2441).

Characteristics	Frequency (%)
**Gender**	
Male	1414 (57.9%)
Female	1027 (42.1%)
**Age in years**	
≤14	108 (4.4%)
15–24	319(13.1%)
25–34	570 (23.4%)
35–44	505 (20.7%)
≥45	924 (37.9%)
Unknown age	15 (0.6%)
**Previous TB treatment history**	
New	1701(69.7%)
Previously treated	254 (10.4%)
Not indicated	486 (19.9%)
**Classification of previously treated**	
Relapse	215 (84.7%)
Treatment failure	24 (9.4%)
Return after default	12 (4.7%)
Other	3 (1.2%)
**Type of specimens**	
Respiratory (sputum)	1895 (75.3%)
Non-respiratory (extrapulmonary)	620 (24.7%)
**Sputum quality**	
Mucoid	973 (38.7%)
Mucopurulent	14 (0.6%)
Purulent	574 (22.8%)
Saliva	263 (10.5%)
Bloody or blood-stained	61 (2.4%)
Not recorded	10 (0.4%)
**Health institutions**	
Government	889 (36.4%)
Private	1155 (47.3%)
Charity	397 (16.3%)

### Xpert test results

A total of 2515 samples were analyzed using the Xpert MTB/RIF molecular assay over the one year study period; the mean number of samples processed each month was 210 (range 36 to 296). Of these, 2274 (90.4%) had successful Xpert test results. Among the successful test results, MTB positivity was 18.2% (95% CI: 16.8–19.7). Of the latter, the percentages of RIF resistance and RIF resistance indeterminate were 4.8% (95% CI: 2.9–7.0%) and 1.0% (95% CI: 0.2–2.2%), respectively. The proportion of unsuccessful test results of the initial testing was 9.6% (Table 3).

**Table 3:** Xpert MTB/RIF results at National TB Reference Laboratory of Ethiopia, January 2018 –December 2018, N = 2515.

Xpert MTB/RIF test outcomes		Frequency (%)
Successful test results, n = 2274	MTB positive	415 (18.2)
	Rifampicin resistant	20 (4.8%)
	Rifampicin indeterminate	4 (1.0%)
	Rifampicin susceptible	391 (94.2)
Unsuccessful test results, n = 241	Error	225 (8.9)
	Invalid	1 (0.04)
	No result	15 (0.6)

### Xpert retesting results

Among the 241 unsuccessful test results (error, invalid, and no result), 232 (96.3%) were retested using leftover SR-treated or newly collected samples. The median time to perform the retesting was 122.5 (IQR, 88–279.8) minutes. In the retested group, the percentage of MTB positivity was 17.9%. Thus, additional 35 MTB cases were detected by retesting initially unsuccessful tests. Two (5.7%) of the retested positives were RIF resistant while one was RIF indeterminate. Thirty-six (15.5%) of the retested specimens did not yield any result (Table 4). Out of the 36 unsuccessful primary retests, a secondary retest was conducted on 27 samples (75%) and results available for 22 (81.5%) of them. The secondary retesting identified five additional MTB cases; all of them were RIF-susceptible and 17 negative results. Thus, in the secondary retest, five additional errors were recorded. Two of the five errors were retested for the third time; one of them was RIF susceptible TB and the other was negative for TB. Therefore, overall 42 MTB cases (9.2% of the total detected cases) were detected by various levels of retesting.

**Table 4:** Xpert MTB/RIF retest results after initial test yielded unsuccessful test results (error, invalid, and no result), n = 232.

Retesting required	Retested N(%)	Successful retest	Not retested	MTB detected	Rifampicin Resistance			Unsuccessful retest	Error	Invalid	No result
					Detected	Indeterminate	Not Detected				
**Error (n = 225)**	216 (96)	184 (85.2%)	9 (4%)	33 (17.9%)	2 (6.06%)	1 (3.03%)	30 (90.9%)	32 (14.8%)	30 (13.9%)	0 (0.0%)	2 (0.93%)
**Invalid (n = 1)**	1 (100)	1 (100%)	0 (0.0%)	0 (0.0%)	0 (0.0%)	0 (0.0%)	0 (0.0%)	0 (0.0%)	0(0.0%)	0 (0.0%)	0 (0.0%)
**No result (n = 15)**	15(100)	11 (73.3%	0 (0.0%)	2 (18.2%)	0 (0.0%)	0 (0.0%)	2 (100%)	4(26.7%)	4 (26.7%)	0 (0.0%)	0 (0.0%)
**Total**	232 (96.3)	196 (84.5%)	9 (3.7%)	35 (17.9%)	2 (5.7%)	1 (2.9%)	33 (91.4%	36 (15.5%)	34 (14.7%)	0 (0.0%)	0 (0.0%)

A total of 261 retests were performed because of unsuccessful tests and unsuccessful retests. Considering the direct reagent cost of the manufacturer (9.98 USD per Xpert MTB/RIF cartridge; the negotiated public sector pricing) [21], about 2,604.78 USD (261*9.98) was per annum. The cost of unsuccessful tests was estimated to be 2,315.36 USD (232*9.98) whereas the cost of unsuccessful retests was estimated to be 289.42 USD (29*9.98) per annum.

### Quality indicators of Xpert MTB/RIF molecular assay

Four hundred fifteen MTB cases were detected from the 2274 specimens tested successfully using Xpert MTB/RIF assay. Therefore, the percentage of annual MTB positivity (Indicator 1) was 18.2%, ranging from 14.3–25.5% across months. Similarly, the percentages of RIF resistant MTB (Indicator 2) and RIF indeterminate MTB (Indicator 3) were 4.8% (ranging 0.0–8.8%) and 1.0% (ranging 0.0–20.0%), respectively (Table 5).

**Table 5:** Quality indicators of Xpert MTB/RIF testing of the National TB Reference Laboratory of Ethiopia, 2018.

Month	Samples Tested N (%)	Indicator 1: MTB positivity rate	Indicator 2: RIF resistance rate	Indicator 3: RIF indeterminate rate	Indicator 4: Error rate	Indicator 5: Invalid rate	Indicator 6: ‘No result’ rate	Indicator 7 (TAT≤24hrs)	Indicator 7 (TAT≤48hrs)
January	269 (10.7)	49 (18.8)	0 (0.0)	0 (0.0)	9 (3.4)	0 (0.0)	0 (0.0)	87 (32.3%)	256 (95.2%)
February	294 (11.7)	50 (18.1)	1 (2.0)	0 (0.0)	18 (6.1)	0 (0.0)	0 (0.0)	159 (54.1%)	283 (96.3%)
March	287 (11.4)	43 (15.9)	3 (7.0)	0 (0.0)	16 (5.6)	0 (0.0)	0 (0.0)	127 (44.3%)	276 (96.2%)
April	257 (10.2)	45 (20.1)	3 (6.7)	0 (0.0)	30 (11.7)	0 (0.0)	3 (1.2)	105 (40.9%)	255 (99.2%)
May	226 (9.0)	38 (19.3)	2 (5.3)	2 (5.3)	28 (12.4)	0 (0.0)	1 (0.4)	167 (73.9%)	222 (98.2)
June	155 (6.2)	21 (15.8)	0 (0.0)	0 (0.0)	21 (13.5)	0 (0.0)	1 (0.6)	123 (79.4%)	155 (100%)
July	238 (9.5)	34 (16.4)	3 (8.8)	1 (2.9)	24 (10.1)	0 (0.0)	7 (2.9)	185 (77.7%)	236 (99.2%)
August	264 (10.5)	35 (15.8)	3 (8.6)	0 (0.0)	42 (15.8)	0 (0.0)	1 (0.4)	202 (76.5%)	264 (100%)
September	212 (8.4)	35 (19.3)	3 (8.6)	0 (0.0)	28 (13.2)	1 (0.5)	2 (0.9)	170 (80.2%)	210 (99.1%)
October	36 (1.4)	5 (14.3)	0 (0.0)	1 (20.0)	1 (2.8)	0 (0.0)	0 (0.0)	36 (100%)	36 (100%)
November	142 (5.6)	35 (25.5)	0 (0.0)	0 (0.0)	5 (3.5)	0 (0.0)	0 (0.0)	119 (83.8%)	141 (99.3%)
December	135 (3.4)	25 (18.9)	2 (8.0)	0 (0.0)	3 (2.2)	0 (0.0)	0 (0.0)	131 (97.0%)	135 (100%)
Total	2515 (100)	415 (18.2)	20 (4.8%)	4 (1.0%)	225 (8.9)	1 (0.04)	15 (0.6)	1611 (64.1%)	2469 (98.2%)

Green: within the target; Yellow: around the margin of the target; Red: above the target

Indicators 1–7: calculated as per the definitions in Table 1

A total of 225 tested specimens were with an error result. The annual error rate (Indicator 4) was 8.9%, varying from 2.2–15.8% across the months. The error rate was higher than the target (<3%) in all months of the year, excluding October and December (Table 5). Overall, 234 total error codes were recorded from 225 error test results. Six error test results had multiple types of error codes; two or three error codes occurred in combination. All happened in combination with error code 5006 i.e. **5006**|1001|1002 (n = 3), **5006**|5007 (n = 2), and **5006**|5017 (n = 1). The error codes or messages were categorized by error types (Table 6). The most common error type was post-run analysis error (92.7%, 217/232). Of the latter, the predominant error code was 5007 (92.6%, 201/217) due to Probe Check failure. Operation terminated errors (2008 and 2014) and run-time errors (1001 and 1002) were also recorded by 4.7% and 2.6%, respectively (Table 6). However, there was no error associated with cartridge loading and self-test.

**Table 6:** Errors that occurred during Xpert MTB/RIF testing in 2018, N = 234 error codes.

Error type	Error code	Error message	# of cases (%)	Possible Causes	Solution
Post-run analysis errors, n = 217	5006	Probe check failed	8 (3.4)*	• An incorrect amount of reagent was inserted into the cartridge • The reagent had bad quality • Fluid transfer failed	Check if: • The reagent was added to the cartridge correctly • Cartridges were stored correctly Re-test using new cartridges
	5007	Probe Check failed	201 (85.9)	• An incorrect amount of reagent was inserted into the cartridge • The reagent had bad quality • Fluid transfer failed • The sample was processed incorrectly in the cartridge	Check if: • The reagent was added to the cartridge Correctly • Cartridges were stored correctly Re-test using new cartridges
	5011	Signal loss detected in the amplification curve	07 (3.0)	• Loss of tube pressure	• Use a new cartridge
	5017	Probe check failed	1 (0.4)	• Cartridge issue	• Use a new cartridge.
Operation terminated errors, n = 11	2008	Abnormal pressure detected	10 (4.3)	• The filter was clogged by debris in the sample • Pressure sensor failed	• Use a new cartridge • Run a cartridge containing buffer only
	2014	Temperature or Heater failure	1 (0.4)	• The heater A thermistor failed	Check: • The ambient temperature • The internal temperature of the instrument
Run-time errors, n = 6	1001	Temperature or Heater failure	3 (1.3)	• A heater component or a related component failed • Environment temperature is too warm • Fan failure	Check: • The room temperature • The functionality of fans and cleanness of filters
	1002	Temperature or Heater failure	3 (1.3)	• The difference between the temperatures of the two thermistors has exceeded the acceptable difference of 5°C.	• Call Cepheid Technical Support

*Six out of eight (6/8) had multiple error codes

Only a single invalid result case was reported in the month of September 2018, which made the annual invalid rate (Indicator 5) 0.04% and it was within the target (<1%). Also, the annual “no result” rate (Indicator 6) was 0.6%, varying from 0.0–2.9% across the months. The ‘no result’ rate (Indicator 7) was higher than the target (<1%) in the month of July 2018 (2.9%). The percentage of test results that were reported within TAT (≤24 hours) of the assay (Indicator 7) was 64.1%, varying from 32.2–100% across the months. However, 98.1% of the tests were reported within 48 hours of TAT, varying from 95.2–100% depending on the months (Table 5).

### Factors associated with Xpert MTB/RIF error results

In bivariate analysis, site of the specimen, instrument module, tester, and tester experience were associated with a high error rate. Respiratory specimens were 1.9 times more likely to have an error result than non-respiratory specimens (*p = 0*.*001*). Samples tested on instrument module A4, B2, B3, C3, and D3 had a statistically significant association with error result (*p<0*.*05*). When results were stratified by tester experience (<2yrs., 2 – 3yrs., and >3yrs.), samples tested by personnel with 2–3 years of experience were 2.3 times more likely to have an error test result than those with >3 years of experience (*p = 0*.*002*). In multivariate analysis, independent risk factors for an error result included instrument module A4 (AOR 64.7; 95%CI: 4.5–435.2, *p = 0*.*002*), B2(AOR 42.8; 95%CI: 3.4–447.9, *p = 0*.*004*), B3 (AOR 13.7; 95%CI: 1.7–407.5, *p = 0*.*013*), C3 (AOR 13.0; 95%CI: 1.7–48.3, *p = 0*.*013*), and D3 (AOR 14.3; 95%CI: 1.9–407.5, *p = 0*.*010*), and tester experience; <2yrs.(AOR 2.1; 95%CI: 1.1–3.7, *p = 0*.*019*) and 2-3yrs.(AOR 2.4; 95%CI: 1.3–4.4, *p = 0*.*003*) (Table 7).

**Table 7:** Risk factors associated with Xpert MTB/RIF error results.

Characteristics		Test Result		Total n (%)	Bivariate Analysis		Multivariate Analysis	
		Error	Other results		COR(95%CI)	P—value	AOR(95%CI)	P—value
Site of specimen	Respiratory	191	1704	1895(75.3)	1.9(1.3–2.8)	0.001*		
	Non-respiratory	34	586	620(24.7)	1.00			
Sputum sample quality	Saliva	35	228	263(14)	1.4(0.9–2.3)	0.107*		
	Mucoid	94	879	973(51.6)	1.0(0.7–1.4)	.959		
	Mucopurulent	0	14	14(0.7)	0.0(0.0-)	.999		
	Bloodstained	7	54	61(3.2)	1.2(0.5–2.8)	.636		
	Purulent	55	519	574(30.5)	1.00			
Instrument module^	A-1	32	283	315(12.5)	6.1(0.8–45.6)	0.077*	6.2(0.8–46.5)	.078
	A-2	6	48	54(2.1)	0.0(0.0-)	.998	0.0(0.0-)	.998
	A-3	19	289	308(12.2)	1.0(0.1–16.7)	.990	1.0(0.1–46.3)	.993
	A-4	41	166	207(8.2)	81.0(5.6–1166.3)	.001	64.7(4.5–435.2)	.002
	B-1	16	265	281(11.2)	0.7(0.1–7.0)	.772	0.5(0.0–4.2)	.629
	B-2	6	295	301(12.0)	54.0(4.2–687.7)	.002	42.8(3.4–447.9)	.004
	B-3	30	231	261(11.3)	14.7(1.9–112.1)	.010	13.7(1.7–407.5)	.013
	B-4	41	194	235(9.3)	1.1(0.1–18.5)	.934	1.1(0.1–48.6)	.934
	C-1	1	54	55(2.1)	6.7(0.8–58.1)	.080	6.6(0.8–47.3)	.087
	C-2	0	51	51(2.0)	3.6(0.5–27.1)	.222	3.4(0.4–46.4)	.240
	C-3	1	53	54(2.1)	13.3(1.8–99.3)	.011	13.0(1.7–48.3)	.013
	C-4	3	2	5(0.2)	3.3(0.4–25.1)	.256	3.4(0.4–46.3)	.248
	D-1	3	227	230(9.1)	1.1(0.1–9.3)	.931	1.0(0.1–4.2)	.966
	D-2	3	3	6(0.2)	7.0(0.9–52.6)	.100	7.1(0.9–43.9)	.058
	D-3	22	81	103(4.1)	11.4(1.5–84.9)	.017	14.3(1.9–407.5)	.010
	D-4	1	48	49(1.9)	1.00		1.00	
Tester	Tester-1	31	368	399(15.9)	2.2(1.1–4.6)	0.030*		
	Tester-2	25	171	196(7.8)	3.9(1.8–8.3)	.000		
	Tester-3	40	459	499(19.8)	2.3(1.1–4.7)	.020		
	Tester-4	6	75	81(3.2)	2.1(0.7–6.0)	.156		
	Tester-5	13	135	148(5.9)	2.6(1.1–6.0)	.030		
	Tester-6	36	263	299(11.9)	3.6(1.8–7.5)	.000		
	Tester-7	64	553	617(24.5)	3.1(1.6–6.1)	.001		
	Tester-8	10	266	276(11.0)	1.00			
Tester experience	< 2 Year (n = 2)	104	1012	1116	2.3(1.4–4.0)	0.002*	2.1(1.1–3.7)	.019
	2–3 Year (n = 4)	105	937	1042	2.2(1.3–3.9)	.004	2.4(1.3–4.4)	.003
	>3 Year (n = 2)	16	341	357	1.00	1.00	1.00	

*Note*: COR; Crude Odds Ratio; AOR, Adjusted Odds Ratio; CI, Confidence Interval; 1.00, Reference

*, Selected variables for multivariate analysis

^, 16 modules GeneXpert instrument

## Discussion

The study presented the use of monitoring quality indicators of Xpert MTB/RIF in initial tests and unsuccessful result retests. Moreover, it demonstrated a method of investigating potential causes of indicators being out of the acceptable limits or targets. All quality indicators were within their targets, with the exception of error rate (Indicator 4) and TAT (Indicator 7). Error rate and TAT were away from the targets; <3% of error rate and 90% of test results report within 24 hours, throughout the year, excluding the months October and December. In these two months, the test statistic was relatively lower than the others.

The success rate of the initial test was 90.4%. The overall MTB positivity rate (Indicator 1) was 18.2%. However, an additional 42 MTB cases were detected following the various level of retesting due to unsuccessful test and retest results. Considering the additional cases, 456 MTB cases detected from the 2495 presumptive TB/DR-TB patients. This figure (Indicator 1, i.e 18.3%) was within the range of MTB positivity rate (13.42–24.61%) reported by various studies in different areas of Ethiopia [13–18]. The observed variation in MTB positivity rate among reports might be linked with the difference in HIV acquisition, health-seeking behavior, geographic location, and TB control effort in the study settings. Additionally, the knowledge status of health care workers (HCWs) towards the diagnostic tool and the clinical practices could affect the positivity rate of the test [22, 23]. The MTB positivity rate (18.3%) recorded by the present study was better than those reported previously by community-based TB prevalence studies [24–26].

The overall initial RIF resistance rate (Indicator 2) was 4.8%. Two RIF resistant cases were detected in 42 MTB cases, which were detected by retesting of unsuccessful test and retest results (test failures). There was a slight variation in RIF resistance rate (4.8%) following retesting of test failures although the difference was not statistically significant. This observation (4.8%) was similar with the WHO estimate for Ethiopia; 5.2% (95% CI: 2.8–8.4) [27] and those reported by Geleta *et al* [13] and Gelalcha *et al* [18]. However, several other studies in Ethiopia had reported higher RIF resistance rate [14–17]. The inconsistency could be due to the difference in a group of patients subjected to Xpert testing and the enrollment of a large number of previously treated TB patients [28]. The current RIF indeterminate rate (Indicator 3) (1.0%) was lower than those reported earlier in Ethiopia [13, 17]. This shows the bacilli load in most clinical specimens was sufficient in yielding adequate DNA for determining RIF resistance.

In agreement with the result of the study, Creswell *et al* [29] reported a 10.6% unsuccessful rate. Furthermore, even higher unsuccessful rate reported by Gidado *et al* [30] and Agizew *et al* [31]. Xpert data source could be a reason for a higher rate than our study. The studies conducted by Gidado *et al* [30] and Agizew *et al* [31] used GxAlert and GeneXpert software(.gxx file format) as the Xpert data source, respectively. These data sources do not differentiate the retest results of test failures rather they consider them as the initial test of a different sample. For example, in our report, combining unsuccessful results of the initial test and retest all together increases the overall unsuccessful results rate to 10.1%, which is similar with the rate reported by Gidado *et al* [30]. We propose quality indicators for a retest to be analyzed separately so that the actual figure of quality indicators for the initial test can be determined. Also, the cost implication and delay in providing valid test results because of test failures should be assessed. Because of unsuccessful results, we lost 2,604.78 USD per annum by considering the direct reagent cost of the manufacturer (9.98 USD per Xpert MTB/RIF cartridge, which is a negotiated pricing). The reagent cost required for 2515 samples test is 25,099.7 USD ($9.98 per a test); however, the test failures increased the required cost to 27,704.8 USD ($11.02 per a test) i.e. 1.04 USD an increment per a test due to test failures. Thus high unsuccessful rate has an impact on the cost of a test and needs to be maintained within a limit. In addition to data sources, factors such as defective modules, staff experience, and cartridge version (G3 vs. G4) could affect the occurrence of unsuccessful results [31, 32]. On the other hand, relatively lower unsuccessful rate reported by Ardizzoni *et al* [32] and Mustapha *et al* [33]. However, laboratory register used as a sole data source for Xpert data and may not capture the initial test outcomes in case of test failures. This could lead to underreporting of unsuccessful results, or else regular supervision may be provided for Xpert facilities under the project.

In our report, the unsuccessful test results were mainly due to error results (93.4%). The annual error rate (Indicator 4) was 8.9%, which is higher than the target (<3%). The high error rate was not identified and resolved timely as the quality indicators have been analyzed using the data only from the LIS, which captures only the final or reported Xpert results. The tester may have done retest from leftover processed samples in case of unsuccessful results until a positive or negative result is achieved, but only the final result was documented on laboratory register and reported via LIS. This caused underreporting of the error rate in routine monitoring of indicators and falsely led to unnecessary confidence about the assay quality. On the basis of this observation, it can be suggested that the data from the GeneXpert instrument software (.gxx file format) along with the LIS or laboratory register could be utilized for analyzing the indicators for the purpose of discovering unreported unsuccessful results.

The most prevalent error was 5007, which is mainly related to the technical issues, i.e., human errors due to non-adherence to manufacturer-recommended procedure during sample processing such as filling reaction tubes with viscous sputum or incorrect sample volumes, and reagent storage condition [20, 30]. This requires improvement on the technical capability of the testers and the storage condition of cartridges. Similarly, a high percentage of 5006, 5007 and 5008 errors were observed from different resource-limited settings [30, 31]. Previous studies revealed that the G3 of the cartridge is associated with high occurrence of unsuccessful results mainly by the signal loss detection error due to loss of tube pressure (Error 5011) [29, 34]; however, Cepheid improved the cartridge deficiency (G4) to reduce errors mainly caused by signal loss error (Error 5011) and G4 version widely available in March 2013. As we used G4 version of the cartridge for the entire tests, the proportion of Error 5011 was low (3.0%) and it looks that the improvement (G3→G4) has limited the incidence of Error 5011 as previously reported [31, 32, 34].

In contrast to published studies [30][31], in our study, the invalid results occurred at a rate of 0.04% (Indicator 5). This shows that there was no specimen associated inhibition of real-time PCR [19, 35]. It also further indicates that the blood cells in specimens tested were not at the level of interfering PCR amplification. Hemoglobin and lactoferrin were reported as PCR-inhibitor in previous studies [36, 37].

Xpert ‘no result’ is commonly associated with the interruption of power supply or lack of the basics of computer use [20]. In this study, ‘no result’ rate (Indicator 6) was 0.6%, which is below the target (<1%). This shows that the Xpert facility has been continuously provided with stable power supply; the power supply backup in case of interruption functions well. In contrary to our finding, Gidado *et al* [30] reported a relatively higher (2.2%) rate of ‘no result’. The difference was probably due to the level of the diagnostic centers in TB laboratories network. In the present case, the laboratory being central or national probably benefited from having a lower incidence of “no results”. However, power interruption remains a challenge at the lower level of the diagnostic centers in resource-limited countries like Ethiopia.

Sixty-four percent of Xpert test results released within the TAT (≤24 hours), but the laboratory targeted 90% of test results within ≤24 hours TAT (Indicator 7). Therefore, the laboratory failed to meet its target. When the TAT extended to ≤48 hours, 98% was attained. Recently, Shiferaw and Yismaw reported 46.2% Xpert tests within targeted TAT in Ethiopia [38]; however, they used shorter TAT (2 hours).

In conclusion, 90.4% of the initial tests were successful. The unsuccessful results rate was high (9.6%); error result was the main contributor. However, the follow-up tests usually resolved the errors and an additional 42 MTB cases detected through retest of failures. Probe check failure was the most frequent error and related to technical and cartridge issues. Instrument modules and tester experience associated with a high error rate. The test results released within TAT was below the target. Hence the present study showed that error rate (Indicator 4) and TAT (Indicator 7) were the two quality indicators that require improvement and continuous assessment. In addition, we illustrated that LIS database or laboratory register along with GeneXpert instrument database (.gxx file format) as the right data source for analyzing the quality indicators in order to avoid underreporting of unsuccessful results. The indicators should be monitored on a monthly basis to identify areas that could compromise quality, investigate possible causes and institute corrective actions in a timely manner. We further proposed the indicators for retesting to be analyzed separately so that the indicators of the initial tests can be determined appropriately. Therefore, the findings of the study can give a good insight into monitoring quality indicators of the assay for other Xpert MTB/RIF laboratories in TB laboratory network of the country.

## Abbreviations

DNA: Deoxyribonucleic Acid
DR TB: Drug Resistant Tuberculosis
EPHI: Ethiopian Public Health Institute
HIV: Human Immunodeficiency Virus
ISO: International Organization for Standardization
LIS: Laboratory Information System
MTB: *Mycobacterium tuberculosis*
NTRL: National TB Reference Laboratory
PCR: Polymerase Chain Reaction
RIF: Rifampicin
TAT: Turnaround Time
TB: Tuberculosis
WHO: World Health Organization
